# Is Carbon Capture and Storage (CCS) Really So Expensive? An Analysis of Cascading Costs and CO_2_ Emissions Reduction of Industrial CCS Implementation on the Construction of a Bridge

**DOI:** 10.1021/acs.est.2c05724

**Published:** 2023-02-02

**Authors:** Sai Gokul Subraveti, Elda Rodríguez Angel, Andrea Ramírez, Simon Roussanaly

**Affiliations:** † SINTEF Energy Research, Trondheim 7019, Norway; ‡ Delft University of Technology, Delft 2628, The Netherlands

**Keywords:** carbon capture and storage, CO_2_ reduction, cost−benefit analysis, cost analysis, CO_2_ emissions

## Abstract

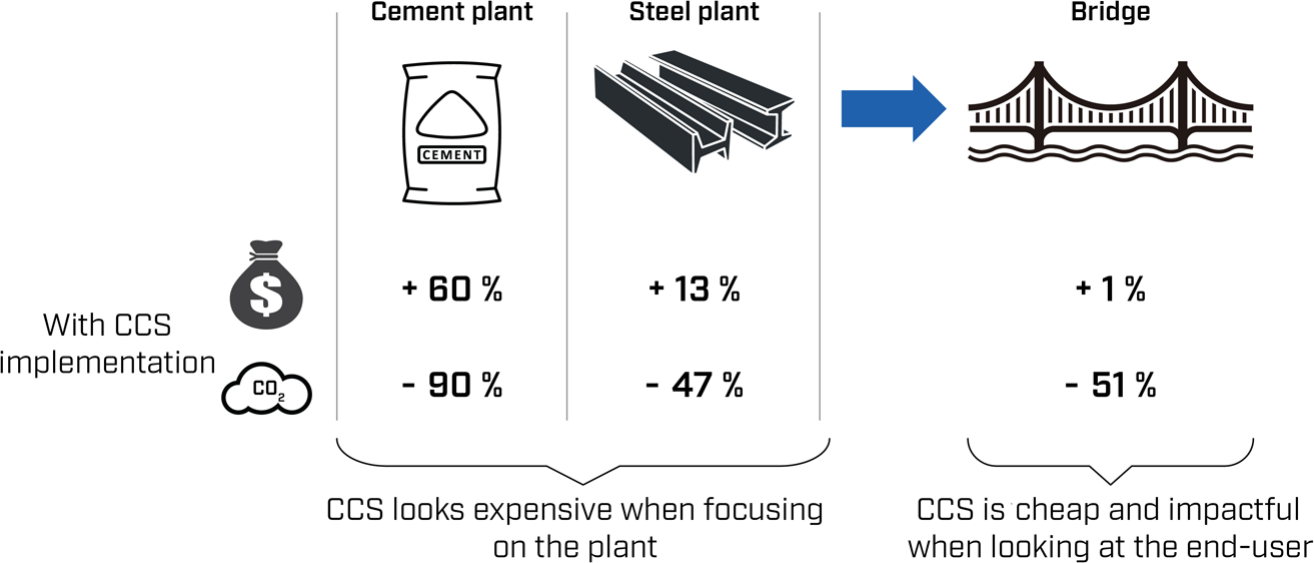

Carbon capture and storage (CCS) is an essential technology to mitigate global CO_2_ emissions from power and industry sectors. Despite the increasing recognition of its importance to achieve the net-zero target, current CCS deployment is far behind targeted ambitions. A key reason is that CCS is often perceived as too expensive. The costs of CCS have however traditionally been looked at from the industrial plant perspective, which does not necessarily reflect the end user’s one. This paper addresses the incomplete view by investigating the impact of implementing CCS in industrial facilities on the overall costs and CO_2_ emissions of end-user products and services. As an example, we examine the extent to which an increase in costs of raw materials (cement and steel) due to CCS impacts the costs of building a bridge. Results show that although CCS significantly increases cement and steel costs, the subsequent increment in the overall bridge construction cost remains marginal (∼1%). This 1% cost increase, however, enables a deep reduction in CO_2_ emissions (∼51%) associated with the bridge construction. Although more research is needed in this area, this work is the first step to a better understanding of the real cost and benefits of CCS.

## Abstract 2

Through the case study of a bridge construction, we demonstrate how carbon capture and storage implementation in industry can enable a deep reduction of CO_2_ emissions at marginal cost for end users.

## 1 Introduction

Meeting the global net-zero target by mid-century to limit global warming to 1.5 °C is necessary to reduce the impacts of climate change significantly.^1^ The deployment of carbon capture and storage (CCS) in the energy and industry sector has been highlighted as critical to cost-efficiently reducing 14% of global CO_2_ emissions.^2^ This is particularly the case in the industry sector (e.g., cement, steel, and chemicals), which is responsible for 45% of global CO_2_ emissions when including indirect emissions.^3^ CCS is one of the few options, especially in the short term, that can significantly reduce industrial CO_2_ emissions.^4^ This is primarily because a quarter of industrial emissions are inherent process emissions from chemical reactions and cannot be avoided by switching to alternative energy sources.^5^ Moreover, there are limited cost-efficient alternatives to fossil fuels for producing high-temperature heat (i.e., a third of industrial energy demand) required in industrial processes. Finally, since industrial facilities are long-term assets, CCS is also attractive as an easily retrofittable solution to mitigate CO_2_ emissions from existing industrial facilities.

Several techno-economic feasibility studies have been carried out to understand the role of CCS in decarbonizing different industries such as cement,^6,7^ iron and steel,^8^ refineries,^9^ chemicals,^10^ pulp and paper,^11^ oil and natural gas processing,^12,13^ and hydrogen.^14^ Beyond these studies, CCS deployment also gained momentum with 20 large-scale CCS projects deployed globally at various industrial facilities currently in operation.^15^ Although the successful demonstration of large-scale CCS deployment is promising for building a carbon-neutral society, the key learnings from the feasibility studies and CCS deployment from industrial sources have highlighted the substantial increase in costs of industrial plants and risks as major challenges. For example, implementing CCS in a cement plant could avoid up to 90% of CO_2_ emissions but would increase the cost of cement production by 65 to 95%, depending on the CO_2_ capture technology.^16^ Since cement, steel, and other chemical industries typically operate at a low-profit margin, the increase in production costs can lead to risks associated with economic repercussions, lower product competitiveness, and producers’ reluctance to deploy CCS in industrial processes.^17^ Although financial mechanisms in the form of fiscal incentives and regulations can initially support CCS deployment in various industries to sustain competitive markets,^17^ the additional cost will eventually be passed over to the end users. Often, the costs evaluated for CCS implementation are only reported on the price of the product(s) of the industrial plant (cement, iron and steel, plastics, etc.), with no information on its impact on products and services consumed by end users in the overall value chain. To provide context, the overall value chain of a product or service is typically made up of three elements: the producer of industrial products (e.g., cement, iron, and steel, etc.), the industrial consumer (e.g., the construction sector consuming cement), and the end user (e.g., people buying a house).^17^ The latter is the reason for the existence of the entire value chain. As the end user will eventually have to incur the additional costs of CCS implementation, it is also essential to evaluate the impact of CCS implementation in industrial plants on end users to fully understand the actual costs of CCS and its true potential in avoiding emissions of products and services.

While most studies in the literature have focused only on assessing the costs of CCS implementation in the industrial processes, only a few studies have examined the cost impact of CCS implementation on the end user by considering an overall industrial value chain. Rootzén and Johnsson, for instance, investigated value chains involving steel and cement production.^18,19^ In one study, the authors examined how reducing CO_2_ emissions by CCS implementation in cement production influenced the costs across the value chain from cement production to the construction of a residential building.^18^ It concluded that the increment in the residential building construction costs is minimal (i.e., 1%) even when the cement production costs doubled with CCS implementation in the cement plant. The authors reported similar observations in another study using the supply of steel to a passenger car as a case study where the cost increment in a passenger car was less than 0.5% even when the cost of producing steel with CCS increased by 35%.^19^

Although earlier studies facilitated the understanding of cost impact on end-user products and services, two significant shortcomings are identified. First, the focus was only on the costs, instead of assessing the impacts of CCS implementation on both, cost and CO_2_ intensity. For instance, if implementing CCS in the cement plant increases the cost of a building by about 10% but, overall, it decreases CO_2_ emissions by only 3%, then the question of the cost–benefit of CCS implementation in reducing CO_2_ emissions arises. Therefore, both costs and CO_2_ intensity must be assessed to fully understand the potential impact of CCS implementation on end-user products. Second, previous research solely considered the impact of CCS implementation in a single industry on a specific end-user product or service (i.e., cement on a house, steel on a car, etc.). However, most end-user products and services rely on multiple products from multiple industries. For example, a house requires a significant amount of cement, steel, and plastics, which are the products of CO_2_-intensive industries.

In this paper, we explore the true potential of CCS implementation in the industry by posing the following question: to what extent does CCS implementation in primary industrial production impact the costs and CO_2_ emission reductions across the overall value chain from industrial plant to end-user products and services? We address this question by considering the construction of a bridge as a relevant example. The bridge as a case study represents a transportation infrastructure commonly used by individuals (i.e., end users) and involves multiple materials such as cement and steel in the construction.

The paper is organized as follows: Section 2 describes the case study and the relevant value chains. Section 3 provides methodology details on costs and life cycle assessment approaches. Section 4 presents the results obtained and discusses their implications. Finally, the key findings are concluded along with some perspectives on how CCS should be perceived in Section 5.

## 2 Case Study

The Lake Pontchartrain Causeway, a beam bridge, located in Louisiana (USA) is here considered as a case study. It is currently the longest beam bridge over continuous water in operation. It is a good representation of a case in which large amounts of primary construction materials are required. To construct the Lake Pontchartrain Causeway, about 225,000 m^3^ of concrete (i.e., 76,487 tonnes of cement assuming 340 kg of cement makes 1 m^3^ of concrete) and 24,209 tonnes of steel (i.e., 2700 tonnes as structural steel and 21,509 tonnes as wire/rod) were required.^20^ As concrete, derived from cement, and steel are produced in different energy-intensive industries, this case study is also representative of a common final product, i.e., beam bridge, produced from more than one material relevant in the context of CCS.

**Figure 1:**
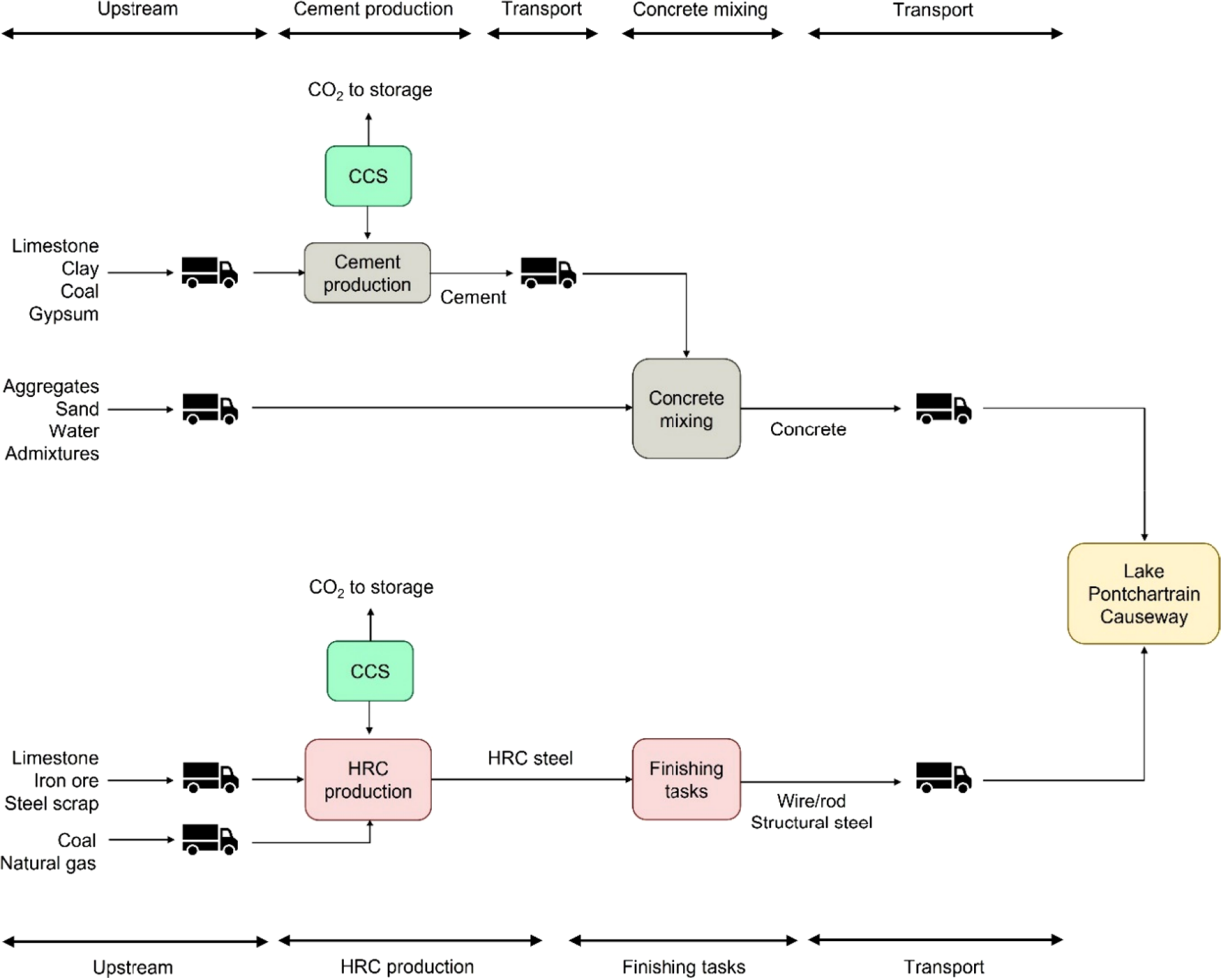
System boundaries of the bridge value chain considered in this study.

Figure 1 presents how the cement and steel value chains are integrated into the construction of the bridge. The cement plant in this case study produced 1.36 Mtonnes of cement per year through a dry kiln process.^6,16^ In a concrete production facility, concrete is produced from cement and other raw materials (agglomerates and water). In this step, only electricity was required as energy input. For this case, it was assumed that the electricity is imported from a power network. We also assumed that cement and concrete were transported by truck. The main source of CO_2_ emissions in the value chain is the primary production facility, i.e., the cement plant.^6,16^ Around half of the onsite emissions in a cement plant are related to coal combustion, while the rest is linked to the calcination reaction in the kiln. The implementation of CCS seeks to reduce these emissions significantly. The other direct or indirect emissions associated with the upstream supply chain, electricity consumption, and transport outside the cement plant are assumed to remain unchanged by implementing CCS. Several technologies can be used to capture the CO_2_ emissions from the cement plant. Here, oxy-fuel capture was considered based on the results from the H2020 CEMCAP project.^6,16^

The steel-to-bridge value chain includes steel (i.e., wire, rods, and structural steel) as the primary product and the bridge as the final product. The steel is produced in an iron and steel plant producing 4 Mtonnes of hot-rolled coil (HRC) per year through a blast furnace route,^8^ followed by additional finishing tasks such as cutting to make different product categories (e.g., wire, rods, and structural steel). In the HRC plant, coking coal and natural gas are used as both feedstock and fuel, while electricity and steam are produced on-site in a natural gas power plant and a boiler.^8^ Steel was assumed to be transported to the bridge construction site by trucks. Implementing CCS on the oxyfuel blast furnace, using MDEA/Pz resulted in an avoidance rate of 47% of the total emissions in the facility. Although the CO_2_ capture unit using MDEA/Pz achieves a 94% capture rate, in this scenario, CCS is implemented only on part of the emissions of the plant (namely, the oxyfuel blast furnace). Although not considered in this study, further emission reduction could be achieved by, for example, using low-carbon hydrogen instead of coking coal as a reducing agent.^21^

Downstream emissions in the bridge value chain, such as those due to bridge usage, operation, and decommissioning were excluded from this analysis.

## 3 Methodology

While more details on estimating the aggregated CO_2_ emissions and costs are provided in the ESI, the following section provides an overview of the approach adopted in this study.

The potential impact of CCS implementation on end user was assessed by carrying out a comparative analysis of products derived from industrial processes with and without CCS implementation. Therefore, CO_2_ emissions and cost estimates along the value chain are presented for two scenarios (with and without CCS). Data for the cement and steel plants with and without CCS implementation were retrieved based on well-known studies.^6,8^ The cost structures related to the concrete mixing and bridge construction were obtained from refs (18, 22).

CO_2_ emissions outside the cement and steel plants were estimated using emission factors from refs (23−26). The overall CO_2_ emissions of the bridge construction were calculated by aggregating emissions from each stage of the value chain, starting from the upstream supply chain involving raw materials extraction and transport to primary production facilities, primary production, intermediate production, transport from primary to intermediate and from intermediate to final production gates, and at the bridge construction site. The emissions in each of these steps are calculated as follows:

- Upstream emissions from the raw material extraction and transport to the primary production facilities in cement-to-bridge and steel-to-bridge chains were accounted for based on emission factors from the literature.^23−25^ The raw materials were assumed to be transported by truck in the upstream supply chain.
- In primary production, the direct CO_2_ emissions were identified from fuel combustion, process emissions (e.g., chemical reactions), and indirect emissions (associated with electricity consumption) when relevant. For the scenario with CCS, most of the CO_2_ produced in the primary production (i.e., steel and cement plants) was captured using post-combustion capture (steel) or oxyfuel (cement), and only the remaining CO_2_ was considered. The CCS implementation resulted in avoiding^1^ 90 and 47% CO_2_ emissions from the cement and steel production facilities, respectively.^6,8^ Notably, the reference study used a CO_2_ emission factor of 262 kg_CO2_ per MWh consumed for the electricity consumption in the cement plant based on the EU 2014 grid.^16^ Since the electricity is produced on-site in the steel plant, the CO_2_ emissions due to electricity consumption are included in the overall emissions.
- Regarding the production of concrete, there are no on-site emissions as this process just involves the mixing of raw materials, but there are indirect emissions associated with the electricity consumption of the process,^27^ which was again assumed to have a carbon emission factor of 262 kg_CO2_ per MWh consumed.^16^ The conversion of HRC into steel involves tasks that generate CO_2_ such as cutting, rolling, and forming.^19^ These additional emissions were included in the analysis using data from Rootzén and Johnsson.^19^
- Emissions associated with transport between facilities were estimated based on truck transport emission factors.^24^
- Finally, onsite emissions at the bridge construction site were calculated as 5% of the total emissions related to the bridge construction without CCS implementation, based on Zhou et al.^26^ The onsite emissions are primarily due to the energy consumed by skilled workers, the use of construction machinery and equipment, generator set, and rebar processing equipment.

The cost of the bridge construction was calculated for the scenarios with and without CCS. This cost, set to approximate the variation cost with CCS implementation, was obtained using a cascading approach where costs were estimated at each stage of the value chain starting with primary production costs, intermediate production costs, transport steps along the value chains, and the construction of the bridge (final product). Here, costs are presented in euro 2018. In case, the cost data in the literature were expressed in a different currency, they were first converted to euro and then updated to 2018. The costs related to each of these steps are estimated as follows:

- The cost of primary products with and without CCS, along with their breakdowns, was directly obtained from recent techno-economic studies on cement and steel production with and without CCS.^6,8^ The investment and operating costs, excluding the raw material and electricity costs, were updated to 2018 using the Chemical Engineering Plant Cost Index. The raw material and electricity costs were calculated based on their annual consumption and unit costs in 2018. Moreover, the CO_2_ transport and storage costs were added to the operating costs for the cases with CCS implementation.
- In the intermediate production stage, the cost of concrete fabrication, including raw materials, except cement, was estimated based on the cost structure reported in Rootzén and Johnsson.^18^ The costs of steel finishing tasks (converting steel into wire, rods, structural steel, etc.) were calculated as a factor of the steel production cost without CCS.^19^ In other words, the cost of wire/rods was equal to the cost of HRC, and the cost of structural steel was 1.23 times the cost of HRC.^19^
- The transport costs were calculated based on unit truck transport prices.^28^ We assume that the raw materials, including cement and steel, are transported 100 km. The transport distance for concrete was assumed 50 km to prevent the cold joint of the concrete.^29^
- The cost of bridge construction was calculated based on four cost components: (1) superstructure costs which include construction material and material manipulation; (2) services and ancillaries; (3) site component costs; and (4) substructure costs.^22^ The costs of concrete and steel with and without CCS from previous steps were used to estimate material costs for the bridge construction.

## 4 Results and Discussions

**Figure 2:**
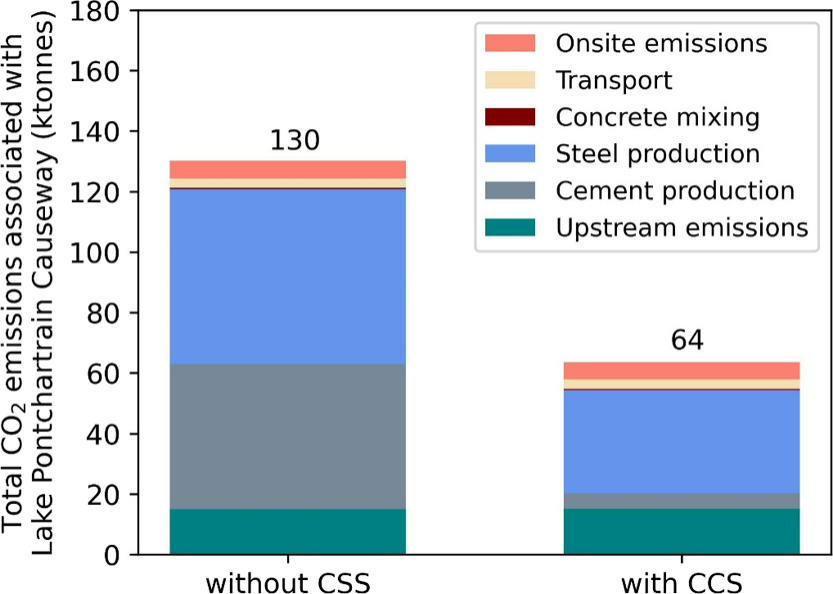
Breakdown of the total CO_2_ emissions for constructing Lake Pontchartrain Causeway with and without CCS scenarios.

This section shows the results of comparative analysis to evaluate the CO_2_ emission reductions and cost increments for bridge construction with and without CCS scenarios. Note that the full results related to the upstream supply chain, cement, concrete, and steel production facilities, along with relevant data are reported in the ESI. Figure 2 illustrates the breakdown of the CO_2_ emissions along the value chain with and without CCS implementation in cement and steel production (also see Table 1). Without CCS implementation, the overall CO_2_ emissions for the bridge construction were about 130 ktonnes, of which upstream emissions (i.e., prior to the cement and steel plants) account for 12%. The CO_2_ emitted in cement and steel plants contribute to 81% of the overall CO_2_ emissions of the bridge. The cement plant alone accounts for 37% of the total CO_2_ emissions (i.e., 48 ktonnes CO_2_). This is primarily due to the emissions arising from the calciner and the rotary kiln because of the combustion of fossil fuels and the limestone calcination process. Steel plant emissions represent 44% of the total CO_2_ emissions (i.e., 58 ktonnes CO_2_), which are due to emissions from the blast furnace, the power plant, coke ovens, lime kilns, the sinter plant, and finishing tasks to produce steel products. Emissions from the concrete plant were negligible (i.e., 0.4 ktonnes CO_2_). The transport emissions resulting from delivering cement, steel, and concrete account for 2% of the overall CO_2_ emissions. The remaining emissions correspond to 4% of the total CO_2_ emissions and are attributed to onsite emissions. CCS implementation in cement and steel plants reduced the overall CO_2_ emissions of the bridge construction by 51% compared to the scenario without CCS.

**Table 1:** Breakdown of Costs and Overall CO_2_ Emissions Associated with the Construction of Lake Pontchartrain Causeway^a^

	without CCS	with CCS
**construction costs (M€)**	**379**	**382**
superstructure costs	160	164
material costs	38	42
steel	11	12
concrete	28	30
manufacturing beam	80	80
concrete placing & deck finishing	3	3
rebar fabrication/placing	14	14
supporting post and form work	18	18
slab waterproofing	6	6
miscellaneous	1	1
services and ancillaries	43	43
site preparation	19	19
substructure	156	156
		
**total CO_2_ emissions (ktonnes)**	**130**	**64**
upstream	15	15
cement production	48	5
steel production	58	34
concrete mixing	0	0
transport	3	3
onsite	6	6

a Bold text is the sum of the items below.

The costs for the bridge construction with and without CCS implementation, along with individual breakdowns, are presented in Table 1. The breakdown of total construction costs includes costs related to the superstructure, substructure, services and ancillaries, and site preparation. The superstructure costs change due to the implementation of CCS in the raw material value chain, all other cost components remain the same. The cost of steel, including the delivery from the steel plant to the construction site, is estimated at 11 and 12 M€ without and with CCS, respectively. This results in an increase in the cost of concrete, including transport to the construction site, from 28 to 30 M€ once CCS is included in the cement value chain. As a result of these small increments, the bridge cost increased only from 379 to 382 M€ once CCS is included in both the cement and steel value chains.

**Figure 3:**
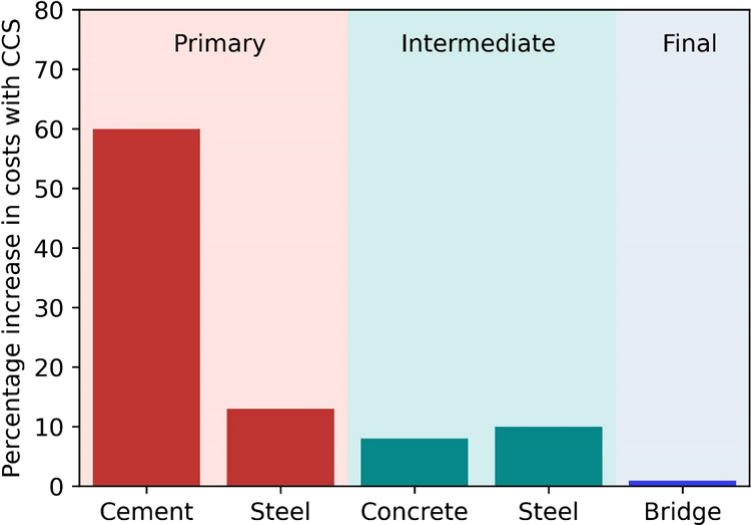
Percentage increase in costs for constructing Lake Pontchartrain Causeway after implementing CCS.

Although the marginal cost increase may appear surprising considering both the significant cost increase that CCS implementation on cement and steel production costs, as well as the considerable share of material in the cost of building a bridge. Figure 3 illustrates that the cascading effect of the CCS cost increases from primary production until the bridge. The production costs of cement and steel (i.e., HRC) increased to 60 and 13% when CCS is implemented, respectively. However, as the share of cement in the concrete formulation is only about 10%, other materials are also required to produce concrete, and the increase in cement costs due to CCS implementation translates to only about an 8% increase in concrete costs. Similarly, considering the additional finishing tasks to convert HRC into different steel products further reduced the cost increment of CCS implementation in steel production to 10%. Combining both material value chains, the costs of raw materials, concrete and steel, for bridge construction are 9% higher with CCS implementation compared to without the CCS scenario. Since the raw materials contribute to only 10% of bridge construction costs, the impact of the increase in costs of raw materials due to CCS implementation on the overall construction costs diminished significantly, as illustrated in Figure 3, to about 1%. Therefore, despite the significant impact on cement and steel costs, implementing CCS in cement and steel production would have had a negligible impact on the construction costs of Lake Pontchartrain Causeway, mainly, because the primary drivers of the overall costs are linked to other construction expenses. In terms of carbon footprint, however, 51% of the direct CO_2_ emissions along the value chain are avoided with CCS implementation in cement and steel plants. Considering these results, it could also be worth considering capture rates higher than 90%^30−32^ as these can be expected to have a marginal cost impact for end users but a significant potential to further reduce emissions.

**Figure 4:**
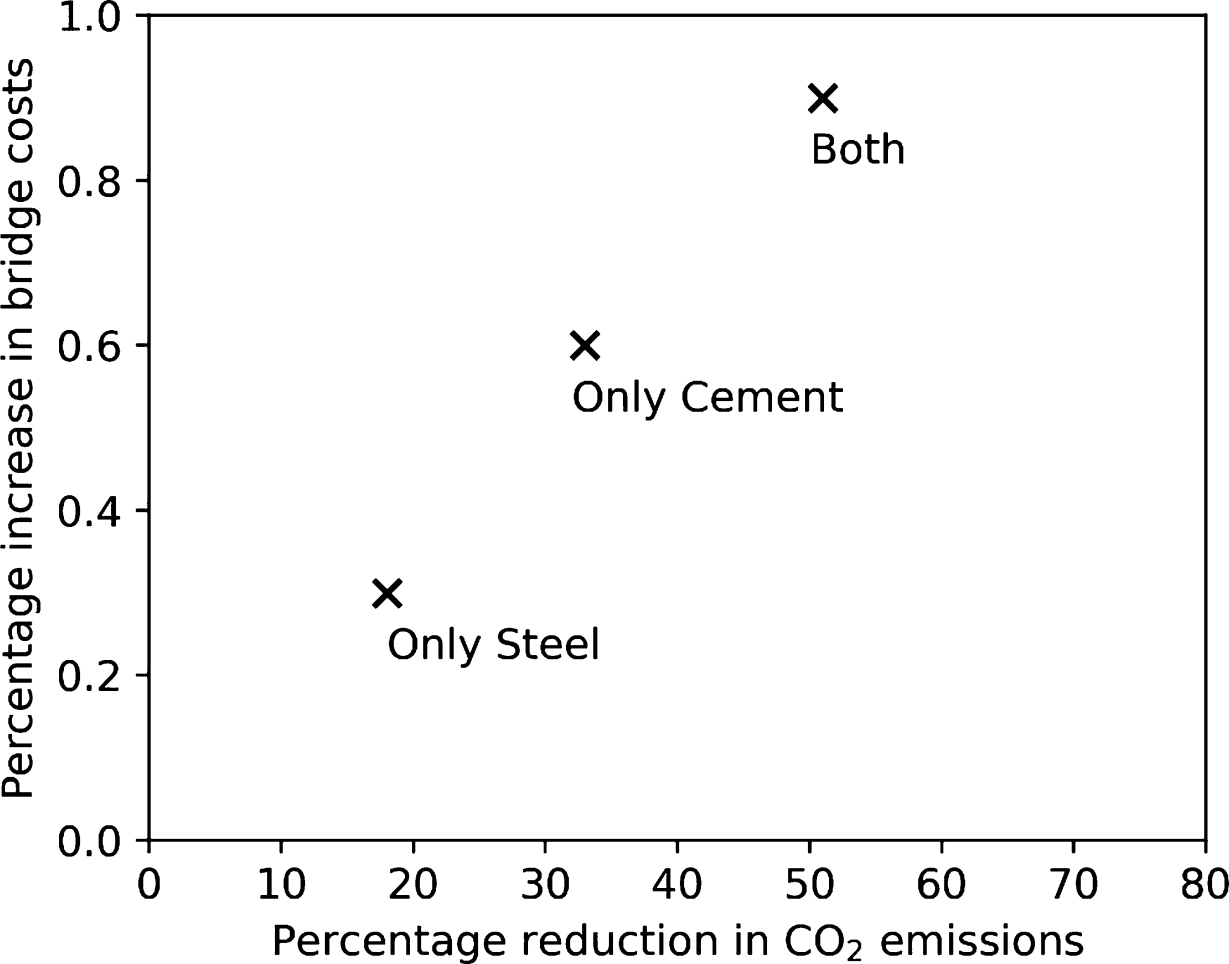
Impact of CCS implementation in the cement plant or the steel plant or both on the percentage increase in bridge construction costs and reduction in overall CO_2_ emissions.

So far, the impact of CCS implementation in both cement and steel plants on the overall costs and CO_2_ emissions linked to the construction of the Lake Pontchartrain Causeway has been investigated. However, it is also important to understand the impact that CCS implementation in each of these industries can have on the cost and CO_2_ emissions of the bridge, as shown in Figure 4. CCS implementation only in cement production yields about 33% emissions reduction while increasing the bridge cost by about 0.6%. CCS implementation in only steel production is responsible for an 18% emission reductions for a bridge cost increase of 0.3%. Thus, in the case of a bridge, CCS implementation in the cement sector is more impactful in terms of CO_2_ emission reductions than CCS implementation in the steel sector. However, it is important to remember that CCS implementation is required in both sectors to deeply reduce the CO_2_ emissions of the bridge and that, in any case, the cost of implementing CCS in both industries has a marginal impact on the cost of the bridge (less than 1%).

In any case, a 1% increase in the bridge construction cost appears highly cost-effective for a 51% reduction in carbon emissions. This positive cost–benefit trade-off emphasizes the strong value of CCS implementation in the cement and steel sectors for this bridge case study. It is worth noting that, even for demonstration projects, which tend to have much higher costs, the impact of CCS implementation in steel and cement would still lead to a marginal cost increase. In addition, the significance of a 51% carbon reduction cannot be ignored – particularly as the cement and steel industry together account for 14% of the world’s CO_2_ emissions.^7,8^ Looking at the impact of CCS in the final value chain can bring new insights to understanding the real costs of CCS in society.

The cost burden/risks associated with CCS can be mitigated by developing strategies to promote coordination and collaboration along the value chain,^19^ promoting public procurement to reduce the risk by creating markets, opening economies of scale, and increasing the demand for the product. Moreover, cities/municipalities supporting complementary policies related to the procurement of low-carbon products can also play an essential role. For instance, this marginal cost increase could be covered through a marginal increase in the toll fee paid by road users to access the bridge or directly by municipalities or more generally the infrastructure owner. Cities and governments have made strong commitments in terms of reduction in 2030 and 2050. Ensuring emissions reduction of such infrastructures through low-carbon materials public procurement could support their 2030 ambitions under the Paris Agreement at a reasonable cost. This could also enable enough demand for low-carbon cement and steel to trigger the further implementation of CCS in the cement and steel sectors beyond the few under construction or already operational CCS facilities from cement and steel such as the Norcem Brevik cement plant (Norway) and the Emirates steel facility in Abu Dhabi (United Arab Emirates).^33^

While more research is needed into the impact of CCS implementation on end-user products and services, this work is the first step to a better understanding of the cost and benefits of CCS. Finally, given the necessity to achieve net-zero emissions by 2050, the approach presented in this study can also be used to gain insight into the cost impact for end users of achieving net-zero products and services through a combination of decarbonization strategies such as energy efficiency, electrification, biomass use, CCS, carbon dioxide removal, etc.

## Supporting Information Available

Methods for calculating costs and CO_2_ emissions of the bridge; a summary of costs and CO_2_ emissions of cement, concrete, and steel production (PDF)

## Footnotes

1 The equivalent CO_2_ avoided is defined as the difference in the CO_2_ emission intensity of the industrial plant without CCS and with CCS, also taking into account all direct and indirect emissions.
