# Supplementary material for: Is Carbon Capture and Storage (CCS) Really So Expensive? An Analysis of Cascading Costs and CO2 Emissions Reduction of Industrial CCS Implementation on the Construction of a Bridge
Source: Environ Sci Technol. 2023 Feb 2;57(6):2595–601. doi: 10.1021/acs.est.2c05724 (PMC9933526; doi:10.1021/acs.est.2c05724)
Supplement: Supplementary file 1 — es2c05724_si_001.pdf [file es2c05724_si_001.pdf]

Supporting Information for

Is CCS really so expensive? An analysis of cascading costs and CO_2 emissions reduction of industrial CCS implementation on the construction of a bridge

Sai Gokul Subraveti[a], Elda Rodríguez Angel[b], Andrea Ramírez[b], Simon Roussanaly[a,*]

[a] *SINTEF Energy Research, 7019 Trondheim, Norway*

[b] *Delft University of Technology, 2628 Delft, The Nederlands*

* Corresponding author: simon.roussanaly@sintef.no

This document of "Supporting Information" consists of 11 pages in total with 12 tables and no figures.

S1 Contents

S1. Methods S3

S1.1. Calculation of CO_2 emissions associated with bridge construction S3

S1.2. Cost estimation S6

S2. Results S10

S2.1. Cement and subsequent concrete production S10

S2.2. Steel and subsequent steel products production S10

S1. Methods

S1.1. Calculation of CO_2 emissions associated with bridge construction

The cradle-to-gate CO_2 emissions associated with bridge construction were aggregated from both concrete and steel value chains as follows:

$$e_{overall} = q_{p,c}(e_{u,c} + e_{p,c} + e_{tp,c}) + q_{i,co}(e_{u,co} + e_{i,co} + e_{ti,co}) + q_{p,s}(e_{u,s} + e_{p,s} + e_{tp,s})$$

where,

$e_{overall}$ corresponds to the cradle-to-gate CO_2 emissions associated with bridge construction (t_{CO_2});

$q_{p,c}$ is the amount of cement (t_{cement});

$e_{u,c}$ are upstream CO_2 emissions related to the raw material extraction and their transport to the cement plant (t_{CO_2}/t_{cement});

$e_{p,c}$ are CO_2 emissions of the cement plant (t_{CO_2}/t_{cement});

$e_{tp,c}$ are transport emissions of cement to concrete production facility (t_{CO_2}/t_{cement});

$q_{i,co}$ is the amount of concrete ($m^3_{concrete}$);

$e_{u,co}$ are upstream CO_2 emissions related to the raw material extraction (excluding cement) and their transport to the concrete plant ($t_{CO_2}/m^3_{concrete}$);

$e_{i,co}$ are CO_2 emissions of the concrete plant ($t_{CO_2}/m^3_{concrete}$);

$e_{ti,co}$ are transport emissions of concrete to bridge construction site ($t_{CO_2}/m^3_{concrete}$);

$q_{p,s}$ is the amount of steel (t_{steel});

$e_{u,s}$ are upstream CO_2 emissions related to the raw material extraction and their transport to steel production facility (t_{CO_2}/t_{steel});

$e_{p,s}$ are CO_2 emissions of steel production (t_{CO_2}/t_{steel});

$e_{tp,s}$ are transport emissions of steel to bridge construction site (t_{CO_2}/t_{steel}).

It is worth noting that HRC is converted to several products and forms of steel (e.g., wire, rod, and structural steel) utilizing some tasks that emit CO_2 [1]. These emissions ($e_{f,s}$) were added to the CO_2 emitted by the steel production plant as follows:

$$e_{p,s} = q_r e_{p,HRC} + e_{f,s}$$

where,

$e_{p,s}$ are CO_2 emissions of the steel product (t_{CO_2}/t_{steel});

$e_{p,HRC}$ are CO_2 emissions of the HRC-steel plant (t_{CO_2}/t_{steel});

q_r is the amount of steel obtained from one tonne of HRC (t_{HRC}/t_{steel}).

It is assumed that one tonne of HRC is converted into one of any steel products, i.e., $q_r = 1$.

The upstream emissions (e_u) were aggregated by taking into account all the emissions related to raw materials extraction and their transport to primary/intermediate production facilities as follows:

$$e_u = \sum_r (e_{u,r} + e_{u,tr})$$

where,

$e_{u,r}$ are CO_2 emissions related to raw materials extraction in the upstream supply chain;

$e_{u,tr}$ are transport emissions related to raw materials in the upstream supply chain.

Table S1: Summary of input data for estimating CO_2 emissions and variable operating costs in cement value chain.

Parameter	Unit	Without CCS	With CCS	Data Source
**Cement production**				
Clay	t/t_{cement}	0.241	0.241	[2]
Clinker	t/t_{cement}	0.737	0.737	[3, 4]
Coal	t/t_{cement}	0.086	0.086	[4]
Electricity	MWh/t_{cement}	0.097	0.207	[3]
Gypsum	t/t_{cement}	0.050	0.050	[5]
Limestone	t/t_{cement}	0.339	0.339	[4]
**Concrete mixing**				
Admixtures	$t/m^3_{concrete}$	0.002	0.002	[6]
Cement	$t/m^3_{concrete}$	0.340	0.340	[6]
Crush aggregates	$t/m^3_{concrete}$	0.950	0.950	[6]
Electricity	$MWh/m^3_{concrete}$	0.005	0.005	[5]
Sand	$t/m^3_{concrete}$	0.900	0.900	[6]
Water	$t/m^3_{concrete}$	0.190	0.190	[6]
**Transport**				
Transport, truck	km	100	100	assumed

Table S2: Summary of input data for estimating CO_2 emissions and variable operating costs in steel value chain.

Parameter	Unit	Without CCS	With CCS	Data Source
Coal	t/t_{HRC}	0.67	0.55	[7]
Iron ore	t/t_{HRC}	1.36	1.36	[7]
Limestone	t/t_{HRC}	0.289	0.249	[7]
Natural gas	GJ/t_{HRC}	0.849	5.045	[7]
Steel scrap	t/t_{HRC}	0.126	0.126	[7]
Transport, truck	km	100	100	assumed

Table S3: CO_2 emission factors of the upstream supply chain.

Parameter	Unit	Value	Data Source
Admixtures	$kg_{CO_2}/t_{admixtures}$	1620	[2, 5]
Clay	kg_{CO_2}/t_{clay}	9.6	[2]
Coal	kg_{CO_2}/t_{coal}	168	[2]
Crush aggregates	$kg_{CO_2}/t_{aggregates}$	5.1	[2]
Electricity, grid	kg_{CO_2}/MWh	390	[2]
Gypsum	kg_{CO_2}/t_{gypsum}	7.2	[2]
Iron ore	$kg_{CO_2}/t_{iron\ ore}$	47	[2]
Limestone	$kg_{CO_2}/t_{limestone}$	4.8	[2]
Natural gas	$kg_{CO_2}/t_{natural\ gas}$	285	[2]
Sand	kg_{CO_2}/t_{sand}	10.9	[2]
Steel scrap	$kg_{CO_2}/t_{steel\ scrap}$	121	[2, 8]
Transport, truck	kg_{CO_2}/tkm	0.084	[2]
Water	kg_{CO_2}/t_{water}	0.3	[2]

Table S4: CO_2 emissions of the cement plant without and with CCS implementation.

CO_2 emissions	Without CCS	With CCS	Data Source
CO_2 generated (before capture) (kg_{CO2}/t_{cement})	626	649	[3]
CO_2 captured (kg_{CO2}/t_{cement})	-	584	[3]
CO_2 emitted (after capture) (kg_{CO2}/t_{cement})	-	65	[3]
CO_2 avoided	-	90%	[3]

Table S5: CO_2 emissions of the steel plant without and with CCS implementation.

CO_2 emissions	Without CCS	With CCS	Data Source
CO_2 generated (before capture) (kg_{CO2}/t_{HRC})	2090	1976	[7]
CO_2 captured (kg_{CO2}/t_{HRC})	-	861	[7]
CO_2 emitted (after capture) (kg_{CO2}/t_{HRC})	-	1115	[7]
CO_2 avoided	-	47%	[7]
Conversion of HRC into steel (kg_{CO2}/t_{steel})	300	300	[1]

Table S6: Summary of input data for estimating transport emissions and costs

Parameter	Unit	Value	Data Source
**Truck transport**			
Distance	km	100	assumed
Distance, concrete delivery	km	50	[9]
Truck emission factor	kg_{CO_2}/tkm	0.084	[2]
Unit transport price	€$_{2018}$/tkm	0.04	[10]

S1.2. Cost estimation

The bridge construction cost estimates were obtained along the value chain with and without CCS scenarios. The key performance indicator, bridge construction cost, comprises superstructure costs, service and ancillaries, site preparation, and substructure costs [11]. The bridge construction cost was first estimated for without CCS scenario as follows:

1. Superstructure costs include the cost of materials such as concrete and steel (24% of the superstructure costs), costs of manufacturing beam (50%), concrete placing (1.7%), deck finishing (0.2%), rebar fabrication/placing (8.5%), supporting post (6%), form work (5.3%), slab waterproofing (3.5%) and other miscellaneous costs (0.8%) [11].

   The cost of raw materials was estimated based on the amount of steel and concrete used as the construction material along with their costs:

   $$\text{Cost of raw materials (€)} = q_{i,co}C_{co} + q_{p,s}C_s$$

   where,

   $q_{i,co}$ is the amount of concrete used in the bridge construction ($m^3_{concrete}$);

   C_{co} is the cost of concrete together with delivery costs (€/$m^3_{concrete}$);

   $q_{p,s}$ is the amount of steel used in the bridge construction (t_{steel});

   C_s is the cost of steel together with delivery costs (€/t_{steel}).

   Based on the cost of raw materials, the other components of superstructure costs were estimated using the percentage contribution of each component towards the total superstructure costs.

2. Based on Kim et al. [11], superstructure costs contribute to 42.28% of the total bridge construction costs. The other cost components, services and ancillaries (11.39%), site preparation (5.10%), and substructure (41.23%) were then calculated based on total bridge construction costs.

For estimating bridge construction costs with CCS, the material costs (e.g., steel and concrete) with CCS implementation were used to estimate the cost of raw materials. The cost of the other elements remains unchanged compared to without scenario.

Estimating C_{co} and C_s:

The concrete cost (C_{co}) was obtained by summing the concrete materials cost, delivery cost, fixed cost, and plant cost as follows:

$$C_{co} = m_c + d_c + f_c + p_c$$

where,

m_c is the concrete material cost (€/m^3);

d_c is the delivery cost from the concrete plant to the construction site (€/m^3);

f_c is the fixed cost of concrete production (€/m^3);

p_c is the plant cost (€/m^3).

It is worth noting that m_c represents 50% of C_{co} [6]. The raw materials for concrete include cement, crushed aggregates, pit run sand, admixtures, etc. The raw material composition in the concrete mix is provided in Table S1. The cost of cement with and without CCS was obtained from Gardarsdottir et. al. [3] and other raw material costs were obtained from Rootzén & Johnsson [6]. Therefore, C_{co}, was calculated directly based on m_c. The delivery cost, d_c, was obtained based on the transport cost model. The fixed and plant costs are obtained using their remaining percentage of share in the concrete cost. While estimating m_c, the transport costs from the cement plant to concrete facility were included in the cost of cement. Except for cement cost, all other cost components remain unchanged without and with CCS implementation.

The steel cost (C_s) was obtained by summing the production cost of steel and delivery costs as shown below,

$$C_s = rC_{HRC} + d_s$$

where,

C_{HRC} is the production cost of HRC (€/t_{HRC});

r is the relative cost factor represented as the ratio of the steel product price (€/t_{steel}) and the HRC price (€/t_{HRC});

d_s is the steel delivery cost from the steel plant to the construction site (€/t_{steel}).

The HRC produced in the steel mill plant is converted into several products of steel (e.g., wire, rod, and structural steel) by utilizing some additional tasks. A relative cost factor (r) is used to represent the differences in each steel product cost based on production costs without CCS [1]. Note that $r = 1$ and $r = 1.23$ was used for converting HRC into wire/rod forms of steel and structural steel, respectively [1]. The production cost of HRC with and without was obtained from the literature [7].

The cost data for cement and steel plants without and with CCS implementation were retrieved from the literature [3, 7] and are provided in Tables S7 and S8. The total production costs were obtained based on annualised CAPEX and operating costs as follows:

$$\text{Production cost}\left(\frac{€}{t_{\text{product}}}\right) = \text{annualised CAPEX}\left(\frac{€}{t_{\text{product}}}\right) + \text{fixed OPEX}\left(\frac{€}{t_{\text{product}}}\right) + \text{variable OPEX}\left(\frac{€}{t_{\text{product}}}\right)$$

The annualised CAPEX and fixed OPEX costs from previous studies were directly updated to $€_{2018}$ using Chemical Engineering Plant Cost Index (CEPCI). The variable operating costs include raw material costs, energy costs, and other miscellaneous costs. In the cement plant, the variable operating costs are incurred due to the consumption of raw meal, coal, electricity, ammonia, and other miscellaneous expenses. The variable operating costs in the steel plant are due to the consumption of iron ore, coal, natural gas, scrap and ferroalloys, fluxes, and other consumables. While some of these cost components were directly updated to $€_{2018}$ based on CEPCI, other components such as iron ore, coal, natural gas, and electricity typically have a wide range of price fluctuations over years. To provide a more accurate estimate, the cost contributions from coal and electricity consumption in the cement plant were calculated based on annual coal and electricity consumption and their prices in 2018 (provided in Table S9). Similarly, iron ore, coal, and natural gas costs in the steel plant were estimated based on their annual consumption and unit prices in 2018. The annual consumption of raw materials is provided in Tables S1 and S2. For CCS scenarios, CO_2 transport and storage costs (e.g., 10 $€_{2018}/t_{CO2}$) are also included in the variable operating costs.

Table S7: Cement production costs without and with CCS implementation.

Parameter	Unit	Without CCS	With CCS
CAPEX	$€_{2018}/t_{cement}$	16	27
Fixed OPEX	$€_{2018}/t_{cement}$	14	20
Raw meal	$€_{2018}/t_{cement}$	3.9	3.9
Ammonia	$€_{2018}/t_{cement}$	0.54	0.54
Miscellaneous	$€_{2018}/t_{cement}$	0.85	0.85
CO_2 avoided cost	$€_{2018}/t_{CO_2}$	-	53
CO_2 capture cost	$€_{2018}/t_{CO_2}$	-	51

Table S8: Steel production costs without and with CCS implementation.

Parameter	Unit	Without CCS	With CCS
CAPEX	$€_{2018}/t_{HRC}$	110	132
Fixed OPEX	$€_{2018}/t_{HRC}$	102	108
Scrap & ferroalloy	$€_{2018}/t_{HRC}$	43	44
Fluxes	$€_{2018}/t_{HRC}$	9	8
Consumables & others	$€_{2018}/t_{HRC}$	10	11

CO_2 avoided cost	€$_{2018}$/t$_{CO_2}$	-	55

164

165

166 **Table S9: Unit prices of raw materials and energy.**

Parameter	Unit	Value	Data Source
Admixtures	€$_{2018}$/kg$_{admixtures}$	1.6	[6]
CO_2 transport & storage	€$_{2018}$/t$_{CO_2}$	10.0	[12]
Coal	€$_{2018}$/t$_{coal}$	90.8	[13]
Crush aggregates	€$_{2018}$/kg$_{aggregates}$	0.02	[6]
Electricity	€$_{2018}$/MWh	62	[3]
Iron ore	€$_{2018}$/t$_{iron\ ore}$	59.1	[14]
Natural gas	€$_{2018}$/GJ	6.5	[15]
Sand	€$_{2018}$/kg$_{sand}$	0.02	[6]

S2. Results

S2.1. Cement and subsequent concrete production

The CO_2 emissions and cost estimation presented in Tables S11and S12 are expressed per tonne of cement and per m^3 concrete, respectively. Moreover, the calculations are based on 340 kg of cement is required to produce 1 m^3 of concrete [6].

Table S10: Key results obtained for cement production.

Parameter	Unit	Without CCS	With CCS
Upstream emissions	kg_{CO_2}/t_{cement}	25	25
CO_2 emitted in cement plant	kg_{CO_2}/t_{cement}	626	65
Cement delivery emissions	kg_{CO_2}/t_{cement}	8	8
Variable OPEX	$€_{2018}/t_{cement}$	19	32
Fixed OPEX	$€_{2018}/t_{cement}$	14	20
CAPEX	$€_{2018}/t_{cement}$	16	27
Total production cost	$€_{2018}/t_{cement}$	49	78
Delivery cost	$€_{2018}/t_{cement}$	4	4

Table S11: Key results obtained for concrete production facility.

Parameter	Unit	Without CCS	With CCS
Upstream emissions (excluding cement production)	$kg_{CO_2}/m^3_{concrete}$	35	35
CO_2 emitted in concrete plant	$kg_{CO_2}/m^3_{concrete}$	2	2
Concrete delivery emissions	$kg_{CO_2}/m^3_{concrete}$	10	10
Cement cost	$€_{2018}/m^3_{concrete}$	18	28
Other raw materials cost	$€_{2018}/m^3_{concrete}$	44	44
Concrete delivery cost	$€_{2018}/m^3_{concrete}$	5	5
Fixed cost and plant cost	$€_{2018}/m^3_{concrete}$	57	57
Total production cost	$€_{2018}/ m^3_{concrete}$	124	134

S2.2. Steel and subsequent steel products production

The CO_2 emissions and cost estimation presented in Table S13 are expressed per tonne of HRC or steel.

Table S12: Key results obtained for steel production (including finishing tasks).

Parameter	Unit	Without CCS	With CCS
Upstream emissions (excluding cement production)	kg_{CO_2}/t_{HRC}	220	223
CO_2 emitted in HRC plant	kg_{CO_2}/t_{HRC}	2090	1115
Conversion of HRC into steel	kg_{CO_2}/t_{steel}	300	300
Steel delivery emissions	kg_{CO_2}/t_{steel}	8	8
Variable OPEX	$€_{2018}/t_{HRC}$	209	236
Fixed OPEX	$€_{2018}/t_{HRC}$	102	108
CAPEX	$€_{2018}/t_{HRC}$	110	132
Total production cost - HRC	$€_{2018}/t_{HRC}$	422	475
Total production cost – wire/rod	$€_{2018}/t_{steel}$	422	475
Total production cost – structural steel	$€_{2018}/t_{steel}$	519	572
Steel delivery cost	$€_{2018}/t_{steel}$	4	4

References

1. Rootzén, J., & Johnsson, F. (2016). Paying the full price of steel – Perspectives on the cost of reducing carbon dioxide emissions from the steel industry. *Energy Policy*, *98*, 459–469. https://doi.org/10.1016/J.ENPOL.2016.09.021
2. Tanzer, S. E. (2022). *Negative Emissions in the Industrial Sector*. Ph.D. thesis. https://doi.org/10.4233/UUID:5CA5FEA0-3322-4B0B-948B-AF2D60DC168F
3. Gardarsdottir, S., de Lena, E., Romano, M., Roussanaly, S., Voldsund, M., Pérez-Calvo, J.-F., Berstad, D., Fu, C., Anantharaman, R., Sutter, D., Gazzani, M., Mazzotti, M., & Cinti, G. (2019). Comparison of Technologies for CO_2 Capture from Cement Production—Part 2: Cost Analysis. *Energies*, *12*(3), 542. https://doi.org/10.3390/en12030542.
4. IEAGHG (2013). *Deployment of CCS in the Cement Industry*; 2013/19. Available online: https://ieaghg.org/docs/General_Docs/Reports/2013-19.pdf.
5. Tanzer, S. E., Blok, K., & Ramírez, A. (2021). Curing time: a temporally explicit life cycle CO_2 accounting of mineralization, bioenergy, and CCS in the concrete sector. *Faraday Discussions*, *230*(0), 271–291. https://doi.org/10.1039/D0FD00139B
6. Rootzén, J., & Johnsson, F. (2017). Managing the costs of CO_2 abatement in the cement industry. *Climate Policy*, *17*(6), 781–800. https://doi.org/10.1080/14693062.2016.1191007.
7. IEAGHG (2013). *Iron and steel CCS study (techno-economics integrated steel mill)*; 2013/04. Available online: https://ieaghg.org/docs/General_Docs/Reports/2013-04.pdf.
8. Tanzer, S. E., Blok, K., & Ramírez, A. (2020). Can bioenergy with carbon capture and storage result in carbon negative steel? *International Journal of Greenhouse Gas Control*, *100*, 103104. https://doi.org/10.1016/J.IJGGC.2020.103104
9. Al-Araidah, O., Momani, A., Albashabsheh, N., Mandahawi, N., & Fouad, R. H. (2012). Costing of the Production and Delivery of Ready-Mix-Concrete. *Jordan Journal of Mechanical and Industrial Engineering*, *6*(2). Available online: http://jjmie.hu.edu.jo/files/v6n2/v6n2.pdf#page=61.
10. Strunge, T., Renforth, P., & van der Spek, M. (2022). Towards a business case for CO_2 mineralisation in the cement industry. *Communications Earth & Environment 2022 3:1*, *3*(1), 1–14. https://doi.org/10.1038/s43247-022-00390-0
11. Kim, K. J., Kim, K., & Kang, C. S. (2009). Approximate cost estimating model for PSC Beam bridge based on quantity of standard work. *KSCE Journal of Civil Engineering*, *13*(6), 377–388. https://doi.org/10.1007/s12205-009-0377-0.
12. IEAGHG (2017). *CO_2 capture in natural gas production by adsorption processes for CO_2 storage, EOR and EGR*; 2017/04. Available online: https://ieaghg.org/exco_docs/2017-04.pdf.
13. *Coal, Australian thermal coal - Monthly Price (Euro per Metric Ton) - Commodity Prices - Price Charts, Data, and News - IndexMundi*. Retrieved 10 June 2022, Available online: https://www.indexmundi.com/commodities/?commodity=coal-australian&months=60&currency=eur
14. *Iron Ore - Monthly Price (Euro per Dry Metric Ton) - Commodity Prices - Price Charts, Data, and News - IndexMundi*. Retrieved 10 June 2022, Available online: https://www.indexmundi.com/commodities/?commodity=iron-ore&months=60&currency=eur
15. Natural gas price statistics, Eurostat (2018). Available online: https://ec.europa.eu/eurostat/statistics-explained/index.php?title=Natural_gas_price_statistics
